# Tourniquet use for civilian extremity hemorrhage: systematic review of the literature

**DOI:** 10.1590/0100-6991e-20202783

**Published:** 2021-01-04

**Authors:** CARLOS YÁNEZ BENÍTEZ, PABLO OTTOLINO, BRUNO M PEREIRA, DANIEL SOUZA LIMA, ANTONIO GUEMES, MANSOOR KHAN, MARCELO AUGUSTO FONTENELLE RIBEIRO

**Affiliations:** 1 - Royo Villanova Hospital, SALUD, General, GI and Acute Care Surgery Department - Zaragoza - Zaragoza - Espanha; 2 - Dr. Sótero del Rio Hospital, Trauma and Emergency Surgery Department - Santiago - Santiago - Chile; 3 - Universidade de Vassouras, Pró reitoria de Pesquisa e Pós Graduação - Vassouras - RJ - Brasil; 4 - Dr. José Frota Institute, Trauma and Emergency Surgery Department - Fortaleza - CE - Brasil; 5 - Lozano Blesa University Hospital, GI, Breast and Acute Care Surgery Department - Zaragoza - Zaragoza - Espanha; 6 - Brighton - Sussex University Hospital NHS Trust, Esophagogastric and Trauma Surgery Department - Brighton - Brighton - Reino Unido; 7 - Catholic University of São Paulo PUCSP-Sorocaba, Discipline of General and Trauma Surgery - São Paulo - SP - Brasil

**Keywords:** Hemorrhage, Shock, Hemorrhagic, Multiple Trauma, Wounds and Injuries, Extremities, Hemorragia, Choque Hemorrágico, Traumatismo Múltiplo, Ferimentos e Lesões, extremidades

## Abstract

**Introduction:**

extremity tourniquet (TQ) use has increased in the civilian setting; the beneficial results observed in the military has influenced acceptance by EMS and bystanders. This review aimed to analyze extremity TQ types used in the civilian setting, injury site, indications, and complications*.*

**Methods:**

a systematic review was conducted based on original articles published in PubMed, Embase, and Cochrane following PRISMA guidelines from 2010 to 2019. Data extraction focused on extremity TQ use for hemorrhage control in the civilian setting, demographic data, study type and duration, mechanism of injury, indications for use, injury site, TQ type, TQ time, and complications*.*

**Results:**

of the 1384 articles identified, 14 were selected for review with a total of 3912 civilian victims with extremity hemorrhage and 3522 extremity TQ placements analyzed. The majority of TQs were applied to male (79%) patients, with blunt or penetrating trauma. Among the indications for TQ use were hemorrhagic shock, suspicion of vascular injuries, continued bleeding, and partial or complete traumatic amputations. Upper extremity application was the most common TQ application site (56%), nearly all applied to a single extremity (99%), and only 0,6% required both upper and lower extremity applications. 80% of the applied TQs were commercial devices, and 20% improvised.

**Conclusions:**

TQ use in the civilian setting is associated with trauma-related injuries. Most are single-site TQs applied for the most part to male adults with upper extremity injury. Commercial TQs are more commonly employed, time in an urban setting is under 1 hour, with few complications described.

## RESUMO

**Introdução:**

o uso de torniquete em extremidades (TQ) aumentou no ambiente civil; os resultados benéficos observados nas forças armadas influenciaram a aceitação por equipes de pré-hospitalar (PH) assim como pela população leiga. Esta revisão teve como objetivo analisar os tipos de TQ de extremidades usados em ambiente civil, local da lesão, indicações e complicações.

**Métodos:**

revisão sistemática foi conduzida com base em artigos originais publicados no PubMed, Embase e Cochrane seguindo as diretrizes do PRISMA de 2010 a 2019. Extração de dados focada no uso de TQ de extremidade para controle de hemorragia em ambiente civil, dados demográficos, tipo de estudo e duração, mecanismo de lesão, indicações de uso, local da lesão, tipo de TQ, tempo de TQ e complicações.

**Resultados:**

dos 1.384 artigos identificados, 14 foram selecionados para revisão com total de 3.912 vítimas civis com hemorragia nas extremidades e 3.522 colocações de extremidades TQ analisadas. A maioria foi aplicado em pacientes do sexo masculino (79%), com trauma contuso ou penetrante. Entre as indicações estavam choque hemorrágico, suspeita de lesões vasculares, sangramento contínuo e amputações traumáticas parciais ou completas. A aplicação na extremidade superior foi o local de aplicação mais comum (56%), quase todos aplicados a uma única extremidade (99%), e apenas 0,6% requereram aplicações nas extremidades superior e inferior. 80% dos TQs aplicados eram dispositivos comerciais e 20% improvisados.

**Conclusões:**

o uso de TQ em ambientes civis está associado a traumas. Os TQs comerciais são mais utilizados, com tempo menor que uma hora de uso e poucas complicações.

## INTRODUCTION

There are still controversies and preconceived myths surround the use of TQs for extremity hemorrhage and its evolution from the military to the civilian setting^1^. The first documented use of a military TQ was in 1674 by Etienne J. Morel^2^, almost 200 years later, in 1864, Joseph Lister described its civilian use to obtain a bloodless surgical field^3^. For centuries, TQs were considered useful for extremity hemorrhage but hazardous as well, mainly when used for long periods of prehospital settings^4^ ^-^ ^6^. Recent publications from the military have proven that TQ use is effective for extremity hemorrhage, achieving a reduction in mortality when applied at the point of injury^1^ ^,^ ^7^. These findings, along with the increasing threat to civilians from random shootings and terrorist attacks in North America and Europe^8^ has generated a growing interest for TQs by the public, law enforcement agencies, and EMS. With the increase in TQ use by civilians, there is also an increase in concerns regarding TQ safety and complications. Although there are many reports from the military settings, reports for civilian TQ application are not sufficient and do not seem to be universally accepted. Most of the reported experience in the civilian setting is focused in large urban areas in the U.S; however, indications for their use and risk of complications are not clearly defined. This systematic review aims to analyze demographics and experience of TQ use for extremity hemorrhage in the civilian setting, describing the mechanism and site of injury, indications for their application, type of TQ applied, and induced ischemia time as well as complications associated with their use.

## METHOD

The authors (CY, MR) performed a search of the English literature on PubMed/MEDLINE, Embase and Cochrane Review databases, using the following query (civilian [All Fields] AND (“tourniquets” [MeHS Terms]) and ((“extremities [MeSH Terms] OR “extremities” [All Fields] OR” extremity” [All Fields]) AND (“tourniquets” [MeSH Terms] OR “tourniquets” [All Fields])). The search was limited to original articles, in humans, published in the English language from 2010 to 2019. The manuscripts included for review had to address reports of civilian TQ use for both traumatic and nontraumatic injuries, indications for use, location of the injury, TQ type, TQ ischemia time, and complications. Military TQ use, studies in the pediatric population, TQ use for orthopedic surgery or elective surgery, junctional TQs, veterinary use, and venomous snakebites TQ use were excluded. 

Data extraction focused on extremity TQ type applied (commercial vs. improvised), and time of application, indication for use, site of application, cause of hemorrhage, and mechanism in trauma cases. Additionally, study type and duration, demographic data, and complications were also considered. Study quality was appraised using the Critical Appraisal Skills Programme (CASP) format^9^. Counts from individual reports aggregated using only those papers that contained the data studied, descriptive statistics of specific parameters performed on the combined data. The Preferred Reposting Items for Systematic Reviews and Meta-Analyses or PRISMA statement followed^10^. 

## RESULTS

**Figure 1:**
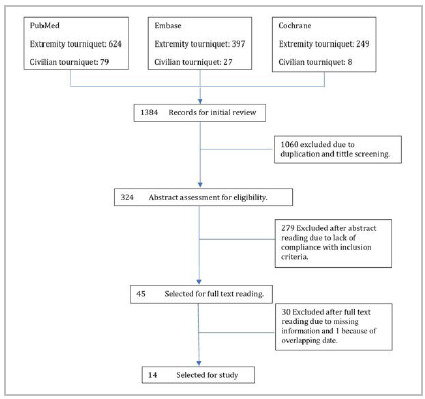
Flow diagram of the systematic review.

The original search included 1384 studies, of which 1060 were excluded due to duplication and title screening (Figure 1). Most of the published literature related to TQ use reported results from the military setting. Among the 324 studies selected, 279 were excluded after abstract review due to lack of compliance with inclusion criteria, and 45 chosen for full-text reading. Thirty publications lacked information relevant to the review, and one was excluded considering it reported date overlapping data for the same center. Finally, fourteen articles published from 2014 to 2019 were selected for full-text analysis and data extraction.

All the selected studies were retrospective, eleven extracted data from hospital records, one from the National Emergency Medical Systems (NEMSIS)^11^, and one from records of the Los Angeles Fire Department (LAFD)^12^. Most of the studies collected data over five years^13^ ^-^ ^21^, thirteen reports were from the United States and one from Canada^15^. Six studies where multi-institutional^14^ ^-^ ^16^ ^,^ ^18^ ^,^ ^22^ ^-^ ^23^, five single-center^13^ ^,^ ^17^ ^,^ ^19^ ^-^ ^20^ ^,^ ^24^ and two were database registries^11^ ^-^ ^12^. 

All the cases of civilian TQ applications for extremity hemorrhage were extracted. In total, 3912 civilian victims were analyzed, accounting for 3522 TQs placed (Table 1). Age was reported in twelve publications with a mean of 38 years^11^ ^-^ ^21^ ^,^ ^23^, gender accounted in eight^11^ ^,^ ^13^ ^-^ ^16^ ^,^ ^19^ ^-^ ^20^ ^,^ ^22^, 2434 (79%) males, and 656 (21%) females. The mechanism of injury was described in all the publications^11^ ^-^ ^23^ but with a heterogeneity of categories. Schroll et al. published cases of penetrating trauma exclusively^22^, five studies included solely trauma cases, and nine distinguished extremity hemorrhage due to traumatic and non-traumatic conditions. Among the commonly reported indications for TQ use were a hemorrhagic shock, suspicion of vascular injury, continued bleeding, and partial or complete traumatic amputations. Among the blunt mechanisms of injury described were extremity trauma due to motor vehicle accidents, open fractures, dog bites, bicycle vs. vehicle, pedestrian vs. vehicle, and crush injuries. Penetrating extremity trauma was mostly gunshot wounds, stabbings, saw, and glass accidents^11^ ^-^ ^22^ ^,^ ^24^. Less frequent trauma mechanisms accounted for were blast injuries (explosive device or industrial accidents)^17^ ^,^ ^19^ ^,^ ^23^. Finally, non-trauma related hemorrhage (arterio-venous hemodialysis fistula bleeding, varicose veins, and bleeding abscesses) was categorized as other^12^ ^-^ ^15^ ^,^ ^18^ ^-^ ^19^ ^,^ ^21^ ^,^ ^24^. In 1381 cases, the authors specified the site of injury and TQ placement, 680 (56%) in the upper extremity and 531 (44%) in the lower extremity^12^ ^-^ ^15^ ^,^ ^17^ ^-^ ^21^. Most were single site or single extremity injuries, only two studies reported 22 (0.6%) patients that required TQ use in both upper, and lower extremities simultaneously^12^ ^,^ ^17^.

**Table 1:** Demographics and TQ types and injury site.

	Published	Period	Duration (years)	Study	SD	N	TQ	Age	M	F	TQ -Type	UE	L E	Both
Passos et al. [16]	2014	2001-2010	10	ER	10	190	8	41	162	28	4 Imp 4 NA	NA	NA	NCR
King et al. [23]	2015	2013	1	ER	1	66	27	33	NA	NA	27 Imp	NA	NA	NCR
Zietlow et al. [21]	2015	2009-2014	6	ER	6	77	77	42	NA	NA	74 C 3 Imp	38	38	NCR
Schroll et al. [22]	2015	2010-2013	4	ER	4	197	197	NA	169	28	157 C 40 Imp	N/A	N/A	NCR
Sanko et al. [12]	2015	2014-2015	2	ER	2	81	81	44	NA	NA	NA	65	17	1
Inaba et al. [13]	2015	2007-2014	8	ER	8	87	87	35	79	8	80 C 7 Imp	62	25	NCR
Kue et al. [15]	2015	2005-2012	8	ER	8	98	98	40	82	16	98 Imp	54	44	NCR
Ode et al. [24]	2015	2012-2013	2	ER	2	56	24	NA	NA	NA	19 C 5 Imp	NA	NA	NCR
Leonard et al. [14]	2016	2009-2014	6	ER	6	95	61	40.5	67	28	61 C	49	46	NCR
El Sayed et al. [11]	2017	2011-2014	4	ER	4	2048	2048	44	1561	487	NA	NCE	NCE	NCR
Scerbo et al. [19]	2017	2008-2016	9	ER	9	306	326	33	258	48	301 C 5 Imp	157	147	NCR
Teixeira et al. [18]	2018	2011-2016	6	ER	6	181	181	34	NA	NA	NA	101	80	NCR
Smith et al. [17]	2018	2010-2018	9	ER	9	238	238	35	NA	NA	205 C 33 Imp	124	115	21
McNickle et al. [20]	2019	2013-2017	4	ER	5	192	69	35	56	13	NA	30	19	NCR
Total				5.7	3912		3522	38	2434	656	897 C 222 Imp	680	531	22

Abbreviations: SD - Study period; TQ - tourniquet; M - male; F - female; Age - years; UE - upper extremity; LE - lower extremity; RS - retrospective study; Imp - improvised-TQ; C - commercial-TQ; NA - not available; NCE - not clearly specified; NCR - no case reported.

Ten studies reported the type of TQ applied, 897 (80%) were commercial devices, most windlass Combat Application Tourniquet - CAT (Composite Resources, Rock Hill, SC), and 222 (20%) improvised TQs^13^ ^-^ ^17^ ^,^ ^19^ ^,^ ^21^ ^-^ ^24^. The improvised devices were frequently blood pressure cuffs, rubber tubing, and home-made devices not specified. The vast majority of the improvised TQs were replaced by commercial windlass TQs by a physician at arrival to the ED, and the duration of TQ use was reported in all but one publication with a mean time of 49 minutes^11^ ^,^ ^13^ ^-^ ^23^.

Complications associated with TQ use were described in eight publications^13^ ^-^ ^15^ ^,^ ^17^ ^-^ ^20^ ^,^ ^22^, and reported with different classifications between the studies (Table 2). Among the most common were nerve palsy, present in 57 of 533 cases (10.7%), rhabdomyolysis in 17 of 164 (10.6%), and thromboembolic events in 23 of 343 cases (6.1%). Other less frequent complications were acute kidney failure (3.8%), compartment syndrome (3.6%), pulmonary complications (7%), cardiac complications (2.8%), and ischemia-reperfusion injuries (2.8%). In the studied population there was only one amputation related to prolonged TQ use in a victim with an upper extremity gunshot wound and 8 hours of TQ related ischemia^13^.

**Table 2:** Tourniquet time, mechanism of injury and complications.

	TQ-T(min)	Mechanism of injury	Complications
Passos et al. [16]	91	Blunt: 2 Penetrating: 2	NCE
King et al. [23]	24	Blast: 27	NA
Zietlow et al. [21]	19	Blunt: 27 Laceration: 21 Stab: 7 Hemodialysis: 5 Fall: 3 Gunshot: 3 Other: 7	NA
Schroll et al. [22]	48	Penetrating: 111	CS: 17 Infection:17 NP: 12 IRI: 7
Sanko et al. [12]	NA	Penetrating: 51 Blunt: 9 AVF: 31 Other: 1	NA
Inaba et al. [13]	103	Stab: 45 Blade: 23 Glass: 18 Other: 42	CS: 2 ARF: 2 Bleeding: 1 HF: 1 Shock: 1 TRA: 1
Kue et al. [15]	15 (NCE)	Penetrating or stabbing: 66 Blunt: 7 Medical: 23	NP: 11 VC: 1
Ode et al. [24]	72	Traumatic laceration: 5 MVA: 5 Gunshot: 4 AVF: 4 Open fracture: 4 Stabbing: 2 Machinery injury: 2 Varicose vein:1	NTRC
Leonard et al. [14]	21	Blunt: 31 Penetrating: 24 FAV: 6	Fasciotomy: 6 Rhabdomyolysis: 2 IRA: 2
El Sayed et al. [11]	41	Stabbing/Accidental cutting: 319 Fall: 269 MVA: 180 Stab: 149 Blunt: 145 Machine accident: 145 Motorcycle accident: 103 Gunshot: 73 Gunshot/Accidental shooting: 45 Pedestrian traffic accident: 39 Bites: 17 Others: 52	NA
Scerbo et al. [19]	21	Blunt: 52 Penetrating: 86	CS: 2
Teixeira et al. [18]	77	Blunt: 81 TxA: 35	Infection: 25 TEE: 13 PC: 13 CC: 5
Smith et al. [17]	23	Penetrating: 176 Gunshot: 54 Knife/saw: 30 Sharp object/glass:41 Animal bite bites: 2 Blunt: 62 MVA: 31 Pedestrian vs. Vehicle: 19 Crush injury: 6 Fall: 2 Bicycle vs vehicle: 2 Blast: 2	NP: 34 Infection:19 CS: 14 ARF: 10 TEE: 8 IRI: 5
McNickle et al. [20]	79	Penetrating: 40 Blunt: 29	Rhabdomyolysis: 15 ARF: 4 CS: 1
Total	Mean time: 49 min.		Rhabdomyolysis: 17/164 (13%) NP: 57/533 (10.7%) Fasciotomy: 6/61 (9.8%) PC: 13/181 (7%) TEE: 21/419 (5%) ARF: 18/455 (4%) CS: 36/917 (3.9%) CC: 5 /181(2.8) IRI: 12/435 (2.8%) Bleeding: 1/87 (1.1%) HF: 1/87 (1%) Shock: 1/87 (1%) TQ-RA: 1/87 (1%)

Abbreviations: TQ-T - tourniquet time; NCE - not clearly specified ; NA - not available; CS - Compartment syndrome; ARF - acute renal failure; HP - hepatic failure; TQ-RA - tourniquet related amputation; VC - vascular complication; IRI - ischemic-reperfusion injurie; TEE - trombo-embolic event; NTRC - no tourniquet related complications; PC - pulmonary complications; CC - cardiac complications; AVF - arteriovenous hemodialysis fistula; MVA - motor vehicle accident; TxA - traumatic amputation; Mangled extremity.

## DISCUSSION

Catastrophic extremity hemorrhage due to trauma in the civilian setting, uncommon until the last two decades, has increased due to active shooter events and terrorist attacks. In 2014 the Texas State University and the FBI reported 160 active shooter events from 2000 to 2013, with 1043 victims killed or wounded^25^. These types of events and increasing threat to civilians has influenced the public`s interest in extremity TQs. During the Sandy Hook Elementary School shooting in 2012, 26 people were shot dead by a 20-year-old man, using a semi-automatic carbine. Following this event, a committee of experts assembled by the American College of Surgeons (ACS), under the leadership of Lenworth Jacobs MD, FACS discussed how to enhance survivability from mass casualty shootings^26^ ^-^ ^27^. This committee’s report known as the Hartford Consensus, emphasized on the THREAT acronym, which stands for T (threat suppression), H (hemorrhage control), RE (rapid extrication), and T (transport to definitive care)^28^, hemorrhage control achieved by local pressure, hemostatic dressings and, if necessary, TQ use. This consensus set the basis for the “Stop the Bleed” campaign, increasing the public awareness of TQs use for extremity life-threatening hemorrhage, advocating point of injury TQ use, and developing a training program for first responders. The purpose was to prepare the young public to save lives in cases of severe bleeding. We consider this campaign appropriate, seeing that of the 3522 TQs applied, 79% were young males with a mean age of 38 years old. It was also noticeable that those who required TQ placement for non-traumatic bleeding were frequently older patients in comparison with the trauma group. 

Our review reveals that the most common cause of hemorrhage requiring TQ application is trauma. Of the studies analyzed, six focused on trauma related TQ use^16^ ^-^ ^20^ ^,^ ^23^, one solely described penetrating trauma^17^, and eight considered both trauma and non-trauma related extremity TQ use, the second referred to as non-traumatic or medical. These less frequent causes of TQ application included hemodialysis arterio-venous shunt bleeding, chronic wounds and varicose vein ruptures^11-15,21,23, 24^. The specific type of trauma that required TQ application resulted uncertain due to the diversity in the trauma categorization in the different studies and should be interpreted carefully. Penetrating trauma was the most common type of injury, among which were gunshot wounds, stabbings, saw, and glass accidents. Blunt trauma followed with motor vehicle accidents, open fractures, bicycle vs. vehicle, pedestrian vs. vehicle, and crush injuries are among the reported mechanisms^11^ ^-^ ^22^ ^,^ ^24^. Blast injuries, either by explosive devices or industrial accidents, were the lowest trauma mechanism reported^17^ ^,^ ^19^ ^,^ ^23^. Likewise, non-trauma related hemorrhage referred to as other categories in the studies was low as well^12^ ^-^ ^15^ ^,^ ^18^ ^-^ ^19^ ^,^ ^21^ ^,^ ^24^. The indications for TQ application was not specified in all the studies, among those reported were life-threatening extremity hemorrhage, traumatic limb amputation, mangled extremity, industrial accidents, crush injuries, blast injuries, multiple bleeding sites in a single patient, multiple-victim scenarios, outdoor wilderness accidents with severe extremity hemorrhage, and remote medical assistance^29^ ^-^ ^30^. Interestingly, only one study defined clearly the absolute indications for TQ use as cases of traumatic amputation or extremity vascular injuries, and relative indications as documented significant blood loss at the scene, major musculoskeletal, or soft tissue injuries^19^. Our findings reflect the lack of uniform guidelines for TQ application in different trauma systems in the U.S., both in urban and rural regions. 

In their study of 2017, El Sayed et al. analyzed data from a U.S. national database of 48 states and territories and estimated an incidence of 0.2 TQ applications per 1000 EMS activations^11^. Smith et al. reported a TQ application increase in New Orleans, from 2.2 per 1000 trauma activations in 2010 to 44.9 per 1000 activations in 2018^17^. Our review unveils an increasing trend in TQ use in urban areas (Houston, Los Angeles, New Orleans, Boston, Las Vegas)^11^ ^-^ ^13^ ^,^ ^15^ ^,^ ^17^. However, this trend of prehospital TQ use seems to be mainly in large cities or urban areas with well-developed trauma systems. Perhaps, these findings are not alike in rural settings; this is relevant considering the differences in health resources and transport time. Of the studies analyzed, two mention TQ use in rural areas^11^ ^,^ ^14^, and only El Sayed et al. considers the relationship between urbanicity and TQ use, reporting a total of 2.048 documented TQ applications in 83,936,070 EMS activations. In their study, 86.4% were in urban or suburban areas and 13.6% in rural areas or the wilderness^11^. The small number of studies considering TQ use in areas with low population density warrant careful interpretation of data regarding TQ use in civilian the setting. 

Individual extremity TQ application was the most frequent, obtaining bleeding control with a single device. Only two studies described injuries requiring both upper and lower extremity TQs on the same patient, and these were only 22 (0,6%) of the reported^12^ ^,^ ^17^. This pattern seems to be usual in civilian settings, different from the military, where multiple extremity injuries on the same victim seem to be more common, requiring more than one device. Our analysis showed that upper extremity was the injured site in 56% of the applications, and lower extremity 44 %, that reveals the importance of upper extremity TQ application training in the civilian educational programs. Although King et al. do not scrutinize the amount of upper or lower extremity affected in his study of the Boston Marathon bombing^23^, we work out from his description that they were mostly lower extremity. The Boston Marathon attack was the first terrorist act in the U.S. that caused multiple lower extremity blast injuries with lower extremity traumatic amputations (LETA); none of the victims had a commercial TQs applied^31^. Instead, 27 improvised TQs were used, most ineffective, and required replacement by commercial devices at arrival to the ED^23^. Despite the differences of military settings with civilian mass casualty incidents, military experience have proven beyond a doubt that improvised TQs are rarely effective in stopping extremity bleeding unless they follow the principles of a windlass device, and commercially available devices can reduce death rates from exsanguinating extremity injuries, like the ones seen in the Boston event^32^ ^-^ ^33^. Currently, several commercial TQs are available to civilians, and experimental studies on volunteers and mannequin models have tested their characteristics and ease of application^25^ ^,^ ^34^. Commercial TQs can be classified based on their mechanism as windlass, ratcheting, pneumatic or elastic TQs. In the studies analyzed, the most common commercial TQ applied was the CAT^13^ ^,^ ^19^ ^,^ ^24^, there are no reports in the reviewed studies of commercial ratcheting, pneumatic, or elastic devices. One of the possible explanations for this finding is that the first version 1.0 of the Stop the Bleed course direct attention to the CAT TQ, and the newer version 2.0 released in 2019 includes several commercial TQs, not only windlass but also ratcheting devices^29^. Some of the reviewed studies mention the use of prehospital pneumatic devices and blood pressure cuffs in the field, the emergency department, and in the operating room^13^ ^,^ ^15^ ^-^ ^16^ ^,^ ^24^. Although improvised TQs are considered controversial, and sometimes ineffective, in some cases they can even contribute to blood loss. Despite these facts the results of improvised TQ use are unclear, Schroll et al. reports in his multicenter study, 197 TQs applied, 40 (20.3%) improvised, and none of these had a higher rate of complications, amputations or death when compared with the ones who received a commercial device^22^. The authors consider that bystanders should learn how to improvise a windlass tourniquet when a commercial device are not available. With independence of the device applied, all TQs, commercial or improvised, require continuous practice on regular bases to maintain proficiency and rapid application. 

TQ time was not registered uniformly in all the studies. Most of the studies registered time from TQ placement to ED arrival^17^ ^,^ ^22^ one reported duration of TQ use separately from prehospital transportation time^13^, others reported it as mean TQ placement time^15^ or mean tourniquet time^14^. Given this heterogeneity among the studies in TQ induced ischemia was registry, there is a limitation interpreting its relationship with the complications observed. Our study also encountered confusing results when analyzing complications attributable to TQ use. Ode et al. described no complications related TQ application among his 24 patients; Smith et al., in their series of 127 patients comparing TQ group vs. Non-TQ group does not report differences in fasciotomy rates, nerve palsy, deep vein thrombosis, or other complications^17^ ^-^ ^24^. In contrast, Inaba in his series reports 13 (15%) complications in 87 patients and Kue et al. 2 (2%) complications in 98 patients; in both series, the authors mention that direct attribution to TQ use was not possible due to the nature of the injury^13^ ^,^ ^15^. Other studies reported higher complication rates, Schroll et al., in a series of 197 patients describes that 64 (32.4%) experienced some type of complication after TQ application; compartment syndrome 17 (3.6%), nerve palsy 12 (6.1%) and ischemia-reperfusion injury 7 (3.6%)^22^. In none of the studies, a relationship was established between the time of ischemia and the incidence of complications. Most of studies that described complications with TQ application didn’t rule out the primary injury as the cause. There were no amputations directly related to TQ use, and only Inaba et al. describe a case of an upper extremity amputation due to a shotgun injury to the right elbow with a transected brachial artery referred from another center after 8 hours of TQ application, in this case, the surgical exploration found non-viable muscle in all compartments. Although, in this particular case, TQ resulted in lifesaving, a contributory role in the limb loss due to the prolonged ischemia could not be ruled out^13^. Finally, the present study aims to demonstrate, based on the experience in the use of tourniquets in a military environment, that the knowledge of the indications as well as the complications in patients victims of major extremity bleeding can contribute to the reduction of mortality rates, causing education programs for both health professionals and the lay population, not only in developed countries but also for countries in less favorable socio-economic conditions where access to emergency care tends to be more time consuming, necessary and urgent.

## LIMITATIONS

Recent battlefield experience, mass shooting events, and terrorist attacks influence the expanding use of tourniquets among civilians. Currently, TQs are considered essential first aid equipment for EMS practitioners and first responders in the civilian setting. In cases of extremity hemorrhage in the civilian setting, commercial TQs are more common than improvised devices. Commercial TQs are the best available option, and only in cases where it’s not available, an improvised windlass TQ can be suitable. Of the several devices designed for civilian use, those with mechanical windlass systems are the most commonly applied. More scientific data is in need to support the use of a specific tourniquet over others. Despite the growing use of TQ in the civilian setting in large urban areas in the United States, there is a lack of uniform guidelines for their application.
